# Adaptive *ε*-greedy exploration for stable reconfiguration in next-gen aviation IMA systems

**DOI:** 10.1038/s41598-025-09025-8

**Published:** 2025-10-29

**Authors:** Guodong Li, Zheyan Liu, Wentao Zhang, Xu Li, Tao Zhang

**Affiliations:** 1 School of Software, Northwestern Polytechnical University, Xi’an, 710072 China; 2 The First Aircraft Institute of AVIC-I, Xi’an, 710089 China

**Keywords:** Computer science, Information technology

## Abstract

The next-generation aviation Integrated Modular Avionics (IMA) system adopts an architecture based on container technology, offering higher resource utilization and task configuration flexibility while increasing system reconfiguration complexity. Efficient reconfiguration strategies enhance adaptability and fault tolerance, ensuring stable operation and reduced maintenance costs. However, existing manual and heuristic-based methods struggle to meet current fault tolerance requirements. We propose an embedded container reconfiguration method using a Double Dueling DQN with Adaptive $\varepsilon$-Greedy Exploration (D3QNAE), which incorporates adaptive exploration to efficiently generate stable strategies in complex environments. Experimental results demonstrate that D3QNAE reduces the first feasible solution time by 34$\%$ compared to the best baseline (D3QN) in large-scale deployments (500-task scenarios), while achieving a 15.6$\%$ higher maximum reward value and a 100$\%$ migration impact rate under continuous faults. This method provides enhanced fault tolerance for container-based IMA systems, significantly improving stability.

## Introduction

The continuous advancement of intelligent and informatization processes in aviation equipment has imposed higher requirements on system architectures. However, traditional avionics architectures are plagued with issues such as relatively inflexible static hardware and software configurations, low resource utilization rates^1^ , difficulties in configuration, and challenges in reconfiguration^2^ . To address these problems, the new generation of IMA systems has introduced container virtualization technology^3^ . Container technology is a lightweight virtualization technique^4–6^ known for its fast startup and high deployment efficiency. It operates solely relying on the application’s binary components and libraries^7^ , thereby being lightweight. This technology has already found its way into applications within IMA systems^8^ .

The introduction of container technology into the new generation of avionics systems has fueled research into reconfiguration technologies for new platforms. Reconfiguration is a crucial mechanism for fault tolerance in avionics systems. It refers to the ability to reallocate system resources during runtime, based on system failures or changing requirements, to ensure that the system meets its functional and performance needs across various environments^9^. With the adoption of container technology, the flexibility of reconfiguration at the system software resource level has been significantly improved due to the agile and lightweight nature of container deployment^10^, thus improving the availability and adaptability of the system. However, concurrently, the complexity of reconfiguration issues in the new generation of avionics systems incorporating container technology has increased significantly compared to traditional systems.

Unlike traditional avionics embedded system reconfiguration techniques, the reconfiguration technology in the new generation of IMA systems based on container technology essentially involves reconfiguring the operating containers^11^. Firstly, the reconfiguration problem transcends from a single-objective optimization in traditional approaches to a multi-objective problem that encompasses container resource utilization, system load balancing, and container migration costs^12^. For safety-critical real-time systems, ensuring fault tolerance through resource-optimal scheduling strategies is crucial, particularly when managing task graphs with precedence constraints and transient faults on multi-core platforms, as demonstrated by formal supervisory control approaches^13^. Additionally, the application of container technology introduces more constraints, requiring reconfiguration strategies to ensure no resource contention among containers while satisfying their performance demands. In heterogeneous environments, energy-aware fault-tolerant scheduling techniques must dynamically balance performance and power efficiency, such as standby-sparing with backup-backup overloading to handle transient faults within stringent deadlines^14^. Lastly, the new generation of avionics systems typically features large-scale, highly complex software architectures^15^, where the number and interrelationships of containers become exceedingly intricate. As such, designing reconfiguration strategies must address the complexity of large-scale systems.Luo et al. demonstrated that an improved Q-learning approach-augmented with a simulated-annealing framework and multi-objective optimization-generates higher-quality reconfiguration blueprints with greater efficiency than simulated annealing, differential evolution, and conventional Q-learning baselines^16^. Consequently, the generation of reconfiguration strategies for the new generation of embedded avionics systems emerges as a complex optimization problem with multiple objectives and constraints, rendering traditional methods insufficient to meet the current demands of the system.

Traditional methods for generating reconfiguration strategies in embedded systems generally fall into manual design or heuristic optimization. Manual design depends heavily on expert experience, making it subjective and difficult to scale as system complexity increases. Heuristic algorithms reduce design workload by automatically exploring the solution space, but often focus only on generating feasible configurations without ensuring optimality in terms of key objectives such as load balancing, recovery time, and migration impact^17,18^.Existing approaches face clear limitations in solving multi-objective reconfiguration problems. Dynamic programming trades space for time but becomes impractical with large data volumes^19,20^; branch-and-bound performs well for single-objective problems but fails to address integrated optimization; metaheuristics such as simulated annealing are prone to local optima^21^. Evolutionary algorithms like genetic algorithms and differential evolution yield better solutions but require excessive computation time^22,23^. Reinforcement learning approaches such as Q-learning support fast scheduling in low-dimensional spaces, but often suffer from instability and poor convergence^24^. Hybrid methods, like simulated annealing Q-learning, improve convergence but require many iterations^16^, while policy gradient methods^25^ are sensitive to gradient noise and costly at scale. Biased estimators^26^ and Monte Carlo Tree Search^27^ partially address these issues but still struggle with exploration efficiency and scalability.Khairullah et al. proposed bio-inspired self-healing hardware architectures, integrating biological concepts to enhance fault tolerance and reliability in safety-critical cyber-physical systems and next-generation nuclear power plants^28,29^.Hybrid Fault-tolerant Scheduling and Load-balancing Model (HFSLM) shows that neighboring-VM reservations can seamlessly recover from VM failures while optimizing makespan and utilization in dynamic clouds^30^.Reserved Fault-Tolerance and Ranked Task Scheduling (RFRTS) improves reliability by synchronizing concurrent VMs and swiftly reallocating tasks to standby instances when CPU faults occur^31^.A recent survey clarifies that hybrid proactive, reactive, and resilient strategies-especially those coupling fault tolerance with load balancing and scheduling-outperform conventional single-aspect methods across diverse failure scenarios^32^.The Priority-Based Task Scheduling with integrated replication-checkpointing (PBTS-FT) model attains >60 % higher recovery rates and markedly better resource use than standalone replication or checkpointing techniques^33^.Beyond the cloud core, a hybrid PSO–GWO metaheuristic delivers fault-aware, energy-efficient resource allocation at the edge, cutting delay and battery drain relative to either algorithm alone^34^.

These challenges highlight the need for a scalable, stable, and adaptive solution that can generate high-quality reconfiguration strategies under dynamic and constrained conditions.To address these challenges, We propose a novel method for generating container reconfiguration strategies in embedded systems based on a Double Dueling Deep Q Network with an Adaptive $\epsilon$-greedy Strategy (D3QNAE). The algorithm in this paper explores the use of an adaptive strategy based on the average sequence cumulative reward. It dynamically adjusts the exploration factor $\epsilon$ according to the cumulative reward value information of the agent during the training process.We adopt an adaptive exploration-exploitation strategy based on the average cumulative reward of action sequences. By dynamically adjusting the exploration factor according to the agent’s accumulated rewards during training, the algorithm effectively balances exploration and exploitation, thereby enhancing overall performance.Some details are as follows: (1)We introduce a Double Dueling Deep Q Network with Adaptive $\epsilon$-greedy Strategy (D3QNAE) to resolve multi-objective optimization in container reconfiguration. (2)We propose an adaptive exploration mechanism that dynamically adjusts $\epsilon$ based on average sequence cumulative rewards , enhancing global optimization in large-scale systems. (3)Experimental validation under single and continuous faults (E1-–E3) demonstrates D3QNAE’s superiority over baseline methods (DQN, D3QN, GA, MOPSO), achieving faster convergence and higher solution quality.

## Methods

### Problem definition

In next-generation integrated electronic systems, embedded platforms must address the challenges posed by dynamic resource fluctuations and localized failures. To ensure continuity and real-time performance, the system must support dynamic migration and reconfiguration of container instances. This problem can be formally defined as the task of designing an efficient migration strategy that reallocates affected containers and their hosted applications across processing units, under strict resource and health constraints, thereby maintaining uninterrupted system functionality.All symbols necessary to resolve this issue, along with their respective definitions,are listed in Table 1.

**Table 1:** Symbols and definitions.

Symbol	Definition
*H*	Set of processors (hardware nodes)
*C*	Set of container instances
*V*	Set of software resources within containers
*S*	Health status of resources, $S \in \{0,1\}$
$t_i$	Time consumption of task *i*
$m_i$	Memory consumption of task *i*
$T_{\max }$	Maximum available CPU time on a processor
$M_{\max }$	Maximum available memory on a processor
*CRB*	Container Reconfiguration Behavior tuple: $\langle S_0, S_f, E, P, S_m, T \rangle$
$S_0$	Initial system state before reconfiguration
$S_f$	Final system state after reconfiguration
*E*	Set of faults
*P*	Set of migration actions or priority queue (context-dependent)
$S_m$	Intermediate states during reconfiguration
*T*	State transition relationships
*S*	System state at a given time: $\{S_{node}, S_{con}, S_{app}, Map\}$
$S_{node}$	Status of all system nodes
$S_{con}$	Container instance information
$S_{app}$	Application software information
*Map*	Mapping relationships among hardware, containers, and apps
*ActionSpace*	Set of actions: $\{A_1, A_2, A_3, A_4\}$
$A_1$, $A_2$	Overall container migration actions
$A_3$, $A_4$	Independent application migration actions
*pri*	Application priority level
*L*	Number of episodes in dynamic reward window
$\overline{G}$	Average cumulative reward of recent *L* episodes
$\epsilon$	Exploration factor in adaptive strategy
$rw(s_i, a_i)$	Reward value of executing action $a_i$ in state $s_i$
*LB*	Load balancing metric
*RT*	Reconfiguration time
*MI*	Migration impact rate
$\mu _a$, $\mu _b$, $\mu _c$	Weights for *LB*, *RT*, and *MI* respectively

System resources are modeled as a quadruple $\langle H, C, V, S \rangle$, where *H* denotes the set of processors, *C* the set of container instances, *V* the software resources within the containers, and $S \in \{0,1\}$ represents the health status of the resources. Each processor must satisfy both time and memory constraints, i.e., $\sum t_i \le T_{\max }$ and $\sum m_i \le M_{\max }$. The container reconfiguration strategy is represented as a six-tuple CRB = $\langle S_0, S_f, E, P, S_m, T \rangle$, which includes the initial state, target state, fault set, migration actions, intermediate states, and the state transition relationships.

The migration process must adhere to key constraints: (1) CPU utilization must remain within the available time window; (2) the memory required by the incoming container must not exceed the remaining capacity of the destination processor; (3) the total memory demand of applications within a container must not surpass the sum of the container image and allocated resources. The overall objective is to minimize system interruption and resource overhead caused by migration, thereby enhancing the system’s adaptability and operational stability in dynamic and fault-prone environments.

### Action and state spaces

The strategy of the system for reconfiguring containers based on faults is regarded as an agent in reinforcement learning, which perceives the state of the environment and selects actions based on the current state to obtain feedback from the environment. The state space needs to comprehensively reflect the system’s state at a particular moment. For a given moment, in conjunction with the mathematical model of the embedded system established, the system state is primarily composed of four parts: node resource status, container instance information, application software information, and the mapping relationships among hardware, container instances, and application software within the system. The mathematical expression is given by Eq. (1):

1 $$\begin{aligned} S=\{S_{node},S_{con},S_{app},Map\} \end{aligned}$$

Here $S_{node}$ represents the information from the system node. The system node information $S_{node}$ consists of all the computing nodes $Node_n$ in the current system, where the information of each node includes the node’s identifier, the maximum time scale of the processor *T*, the maximum memory space of the node *M* and the node state *state* indicating the current operational status of the node.

Based on the established migration action model, the design of the reconfiguration action space is formulated as shown in Eq. (2):

2 $$\begin{aligned} ActionSpace=\{A_1,A_2,A_3,A_4\} \end{aligned}$$

Here according to the migration action model, [*A*1, *A*2] represent the overall migration actions 1 and 2, respectively, which involve migrating containers as a whole. [*A*3, *A*4] represent the independent migration actions 1 and 2, respectively, where individual applications within the container instances to be migrated are moved independently to ensure the completeness of system functionality. In this case, the applications running in the container instances to be migrated are stored in a priority queue *P* based on their software priority *pri*, and the independent migration of applications is performed from highest to lowest priority.

### Dynamic exploration strategy

The exploration strategy adopted by the algorithm is an adaptive one based on the average cumulative reward of the sequence. It dynamically adjusts the exploration factor $\varepsilon$ according to the cumulative reward information obtained by the agent during the training process.

The exploration factor $\varepsilon$ and the average cumulative sequence reward are defined as shown in the following Eq. (3):

3 $$\begin{aligned} {\left\{ \begin{array}{ll} \epsilon = \frac{1}{1+log_{2}{(\overline{G}+1)}} & \\ \overline{G}=\frac{1}{L} \sum _{i=e-L}^{e-1}G_i & \end{array}\right. } \end{aligned}$$

Here *L* represents the number of sequences; $\overline{G}$ denotes the average reward of the first L sequences.

### Reward function design

The reward function is defined with respect to the resource constraints and evaluation metrics of container reconfiguration in an integrated electronic system. Given a current configuration $S_i$, execution of a migration action $a_i$ yields a new configuration $S_{i+1}$, and the transition $(S_i, a_i, S_{i+1})$ is assigned:

1. $r=-1$ if $S_{i+1}$ violates any resource constraint, meaning at least one container instance cannot execute correctly (invalid configuration);
2. $r>0$ if $S_{i+1}$ satisfies all resource constraints, in which case the reward equals a weighted composite score of load balancing, reconfiguration recovery time, and migration impact rate, reflecting the overall quality of the feasible configuration.

Based on the above state designs, the reward function is designed as shown in Eq.(4):

4 $$\begin{aligned} rw(s_i,a_i )= {\left\{ \begin{array}{ll} \mu _a*LB+\mu _b* MI+\mu _c*RT (\mu _a+\mu _b+\mu _c=1) \\ -1 & \end{array}\right. } \end{aligned}$$

Here $rw(s_i, a_i)$ represents the reward value for selecting the migration action $a_i$ when the system is in the configuration state $s_i$; *LB* stands for the load balancing metric, which primarily measures the hardware load of the system under the current configuration state; $\mu _a$ represents the evaluation weight of the load balancing metric; *RT* stands for the reconfiguration recovery time metric, where a shorter reconfiguration time indicates faster fault tolerance and recovery efficiency for the system; $\mu _b$ represents the evaluation weight of the reconfiguration recovery time metric; *MI* stands for the migration impact rate metric, which represents the success rate of reconfiguring the task containers for faulty modules during the learning process of the reconfiguration strategy; the higher the number of successfully configured containers, the higher the configuration rate; $\mu _c$ represents the evaluation weight of the migration impact rate metric.

### Algorithm analysis

In embedded systems, the container reconfiguration problem is characterized by high-dimensional state spaces, complex action combinations, and uncertain environment feedback. Particularly under dynamic scenarios such as processor faults or resource imbalance, traditional Q-learning and standard DQN approaches face significant limitations in policy accuracy, convergence stability, and generalization capability. To address these challenges, this paper introduces the Double Dueling Deep Q Network with Adaptive Exploration (D3QNAE) algorithm, which enhances the learning efficiency and deployment performance of reconfiguration strategies.

D3QNAE employs a double-network architecture (Double DQN) to reduce the overestimation bias of Q-value functions and improve learning stability. Additionally, it incorporates a dueling structure (Dueling DQN) that decouples the state-value function from the action-advantage function, enabling more precise evaluation of the system’s current configuration value, even when action effects are marginally different. Furthermore, the adaptive exploration strategy based on average cumulative rewards allows the agent to dynamically adjust its exploration rate: promoting wide exploration in early training stages and focusing on high-value actions in later stages, thus mitigating the risk of local optima.

From a modeling perspective, D3QNAE defines a comprehensive state space that integrates container instances, application software, hardware resources, and their mapping relationships. The action space is dynamically generated in response to detected faults, ensuring that the learning process accurately captures key characteristics of embedded systems, including resource constraints, real-time scheduling, and fault-tolerant migration.

In terms of computational complexity, let *d* be the depth of the network, *p* the number of parameters per layer, *B* the batch size per training iteration, and *T* the total number of training steps. Then, the overall training time complexity is $O(T \cdot B \cdot d \cdot p)$, while inference (action selection) operates at a complexity of $O(d \cdot p)$. By leveraging experience replay and target network synchronization, the algorithm achieves improved sample efficiency and faster convergence. These properties make D3QNAE both scalable and practical for deployment in resource-constrained embedded environments, providing an effective reinforcement learning framework for solving complex container reconfiguration tasks.

Algorithm pseudocode is provided for detailed reference.

**Algorithm 1:**
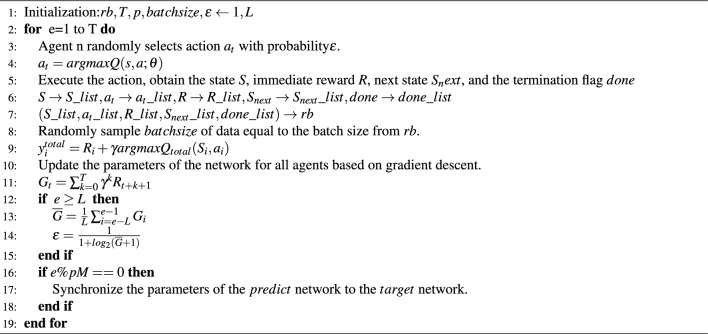
D3QNAE based on the dynamic exploration strategy.

## Results

The experiment was designed to evaluate the performance of reconfiguration strategy generation under known fault scenarios, without involving any fault prediction functionality. Specifically, the objective was to explore how different algorithms respond to various predefined fault patterns in terms of generating effective reconfiguration strategies. To this end, a system resource model was established, and a simulation environment was constructed for an integrated electronic system. In this environment, 15, 100, and 500 task-level application software instances were deployed, each encapsulated in a separate container instance. These setups were defined as experimental environments E1, E2, and E3, respectively. The experiments assessed the performance of different algorithms across these deployment scales, focusing on their ability to generate valid and efficient reconfiguration strategies under varying system loads and fault conditions.

### Fault design

**Fig. 1:**
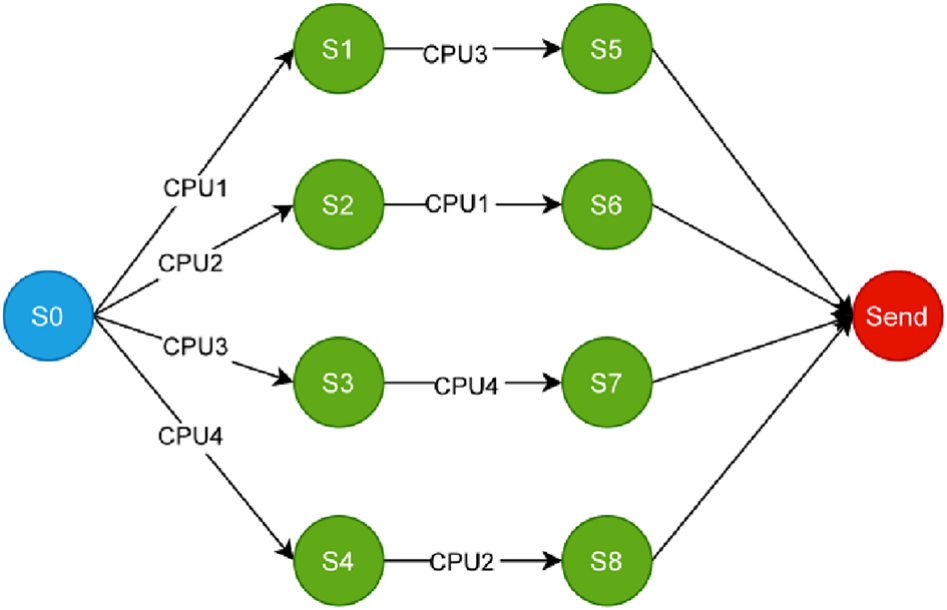
System reconfiguration state transition diagram.

The experimental results are presented in Fig. 1.Faults are injected into the experimental system in a predefined manner, encompassing single faults (S1 to S4) and continuous faults (S5 to S8), with the initial state designated as S0 (represented by a blue node); each fault state corresponds to generating a new reconfiguration state (represented by green nodes), and each fault randomly affects two programs, triggering corresponding container migration strategies to restore normal system operation. The reconfiguration process ultimately reaches the terminal state (*S* *end*, represented by a red node), which can occur under two conditions: first, when there are no remaining faults requiring reconfiguration; second, when the system’s remaining resources are insufficient to perform further reconfiguration actions.

### Single fault experiment

In this experiment, single fault injections were performed in E1, E2, and E3 environments to verify the comparative performance of D2QNAE against algorithms such as D3QN and DQN in terms of learning and solving capabilities for generating reconfiguration strategies in integrated electronic systems of different deployment scales.

For the comparison of algorithm learning performance, the focus was on the iteration of each algorithm’s reconfiguration strategy generation algorithm for 2000 times in E1 and E2 environments, presented as the iteration of the average reward value. Each epoch consisted of an average of 10 iterations, with each iteration involving 100 explorations, where each exploration represented a solution process.

**Fig. 2:**
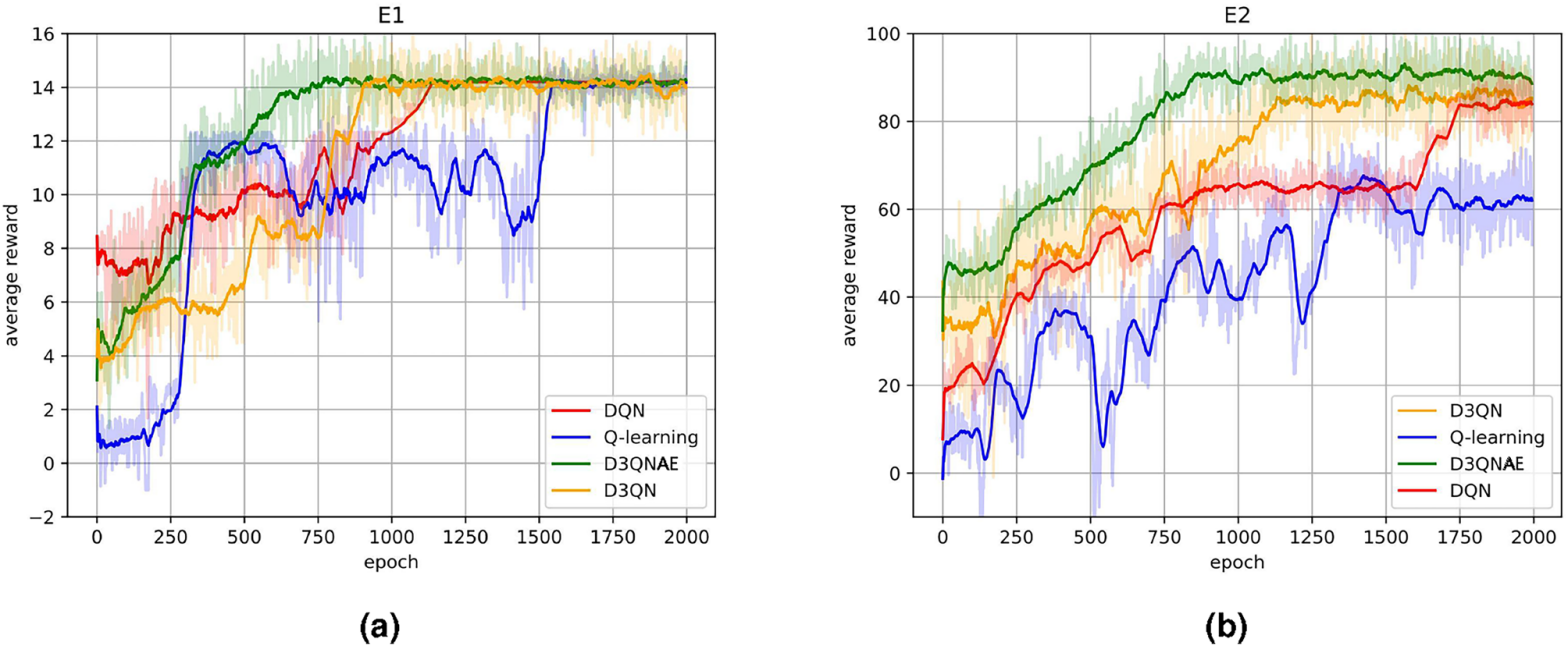
Comparison of reward function reward values during scheduling processes among different algorithms.

As shown in Fig. 2a, algorithms such as D3QNAE and DQN were utilized to solve for reconfiguration strategies under a single fault in Environment E1, where 15 task application software were deployed in the integrated electronic system.

Firstly, all four reconfiguration strategy generation algorithms have converged, indicating that after a certain number of training epochs, their solutions to the problem gradually stabilize, suggesting that under a single fault scenario, all four algorithms can output stable and feasible solutions. During the convergence process, Q-learning showed oscillations around certain local optimal solutions multiple times, which may be due to its greedy strategy struggling to achieve global optimization in complex problems. The trend graphs of D3QN and DQN, which incorporate deep neural networks, exhibited relatively smooth convergence processes, potentially due to the stability brought by experience replay and target networks used during training. D3QN and D3QNAE showed similar trends, but due to the introduction of an adaptive exploration strategy based on average cumulative rewards, D3QNAE was able to find the global optimal solution faster. In contrast, regular D3QN struggled with local optima during convergence, suggesting that dynamically adjusted exploration strategies may have a positive impact on convergence.

As shown in Fig. 2b, a single fault injection test was conducted in E2, the environment with 100 deployed task application software, to observe the efficiency of the algorithms in generating reconfiguration strategies. From the average exploration results, the algorithms with more pronounced upward trends were D3QNAE and D3QN. DQN showed slow growth in the early to mid-stages but a more significant increase later on. Q-learning was able to quickly obtain a higher scoring strategy, but oscillations made it difficult to converge, eventually achieving convergence nonetheless. In smaller-scale system reconfiguration problems, Q-learning can quickly generate feasible solutions but may not guarantee their quality. In contrast, the proposed algorithm D3QNAE excelled in average cumulative reward and iteration trends, achieving optimal solutions and convergence in minimal iterations for solving single fault reconfiguration problems in both small and large-scale systems.

Considering the practical application scenario of emergency fault reconfiguration after a system failure, the comparison of algorithm-solving performance primarily focuses on the time taken by each algorithm to obtain a feasible solution for the first time when generating a single fault reconfiguration strategy in the integrated electronic system environments E1, E2, and E3. The average of 100 such attempts is taken as the final solution time for each algorithm.

**Table 2:** Comparison of first solution times for deep reinforcement learning algorithms across different environments.

Algorithms	E1_15	E2_100	E3_500
Q-learnin	5.56s	10m29s	1h14m28s
DQN	6.08s	2m1s	19m3s
D3QN	4.02s	50.34s	8m3s
D3QNAE	3.08s	35.03s	5m20s

As shown in Table 2, under single-fault scenarios, all selected algorithms can output stable and feasible solutions within 2 hours. Among them, the Q-learning algorithm takes over 1 hour to obtain a reconfiguration strategy for complex systems with large-scale deployed task applications, indicating that the algorithm typically requires some time to learn its own strategy and find feasible solutions through continuous strategy adjustments. The algorithms with shorter solution times are D3QN and DQN, with D3QNAE particularly able to quickly find feasible solutions in both small-scale scenarios with 15 task applications and large-scale scenarios with 500 deployed task applications.

**Fig. 3:**
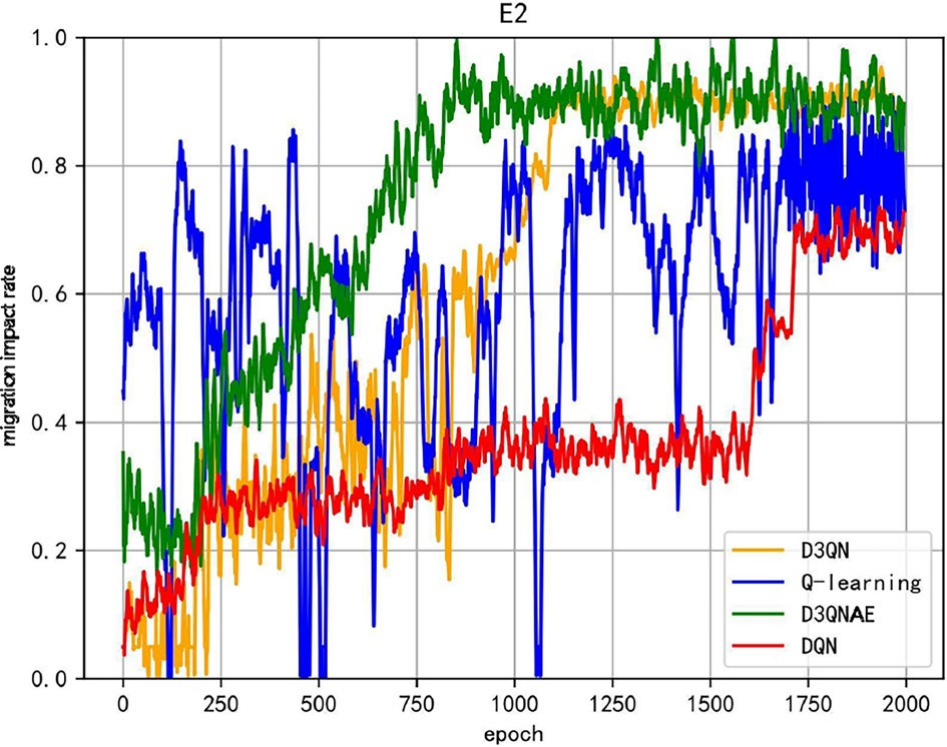
Comparison chart of migration impact rates.

In addition to the existing load balancing and reconfiguration recovery time metrics, the evaluation metrics considered in the algorithm we propose introduce a migration impact rate, aimed at improving the retention rate of container instances for critical task software during reconfiguration. The experimental comparison of the iterative performance of the migration impact rate for reconfiguration strategies generated by different algorithms, taking the E2 environment as an example, is shown in Fig. 3.

The overall trend of the migration impact rate in the reconfiguration strategies generated by the algorithms is similar to the overall average reward function curve. Among them, the Q-learning algorithm exhibits significant oscillations, where a possible scenario for a sharp decline in the migration impact rate is that the reconfiguration strategy generated by the algorithm focuses on minimizing the migration impact rate in the optimization objective, leading the strategy to favor preserving the task applications in the current system, which can compromise load balancing and increase the difficulty of subsequent migration actions in satisfying system constraints. This, in turn, can result in lower-quality or even infeasible reconfiguration strategies, causing sudden drops in the overall average reward function value. In contrast, the D3QNAE algorithm we propose exhibits a relatively smooth migration impact rate curve, potentially due to the stability of the experience replay used during training. This demonstrates a strong ability to resist local optima and balance global and local optimal solutions in multi-objective optimization problems.

In summary, the results of the algorithm learning performance comparison experiments and the algorithm solution performance comparison experiments show that when the system deploys task applications of different scales and a single fault occurs requiring fault reconfiguration, the D3QNAE algorithm we propose can find a good and feasible solution in a relatively short time and gradually optimize the migration impact rate index. Compared to the D3QN and DQN algorithms, the D3QNAE algorithm generates strategies with better quality, and compared to the Q-learning algorithm, it has better global search capabilities.

### Continuous fault experiments

This experiment validates the algorithm learning performance and solution performance of D3QNAE compared to D3QN, DQN, and other algorithms in generating reconfiguration strategies for different-sized integrated electronic system deployment environments by injecting continuous faults into E1, E2, and E3 environments, respectively.

The comparison of algorithm learning performance primarily focuses on the 2000 iterations of the reconfiguration strategy generation algorithms in E1 and E2 environments, presented in terms of the iteration of the average reward value. Each epoch consists of an average of 10 iterations, and each iteration involves 100 explorations, where each exploration is a solution process.

**Fig. 4:**
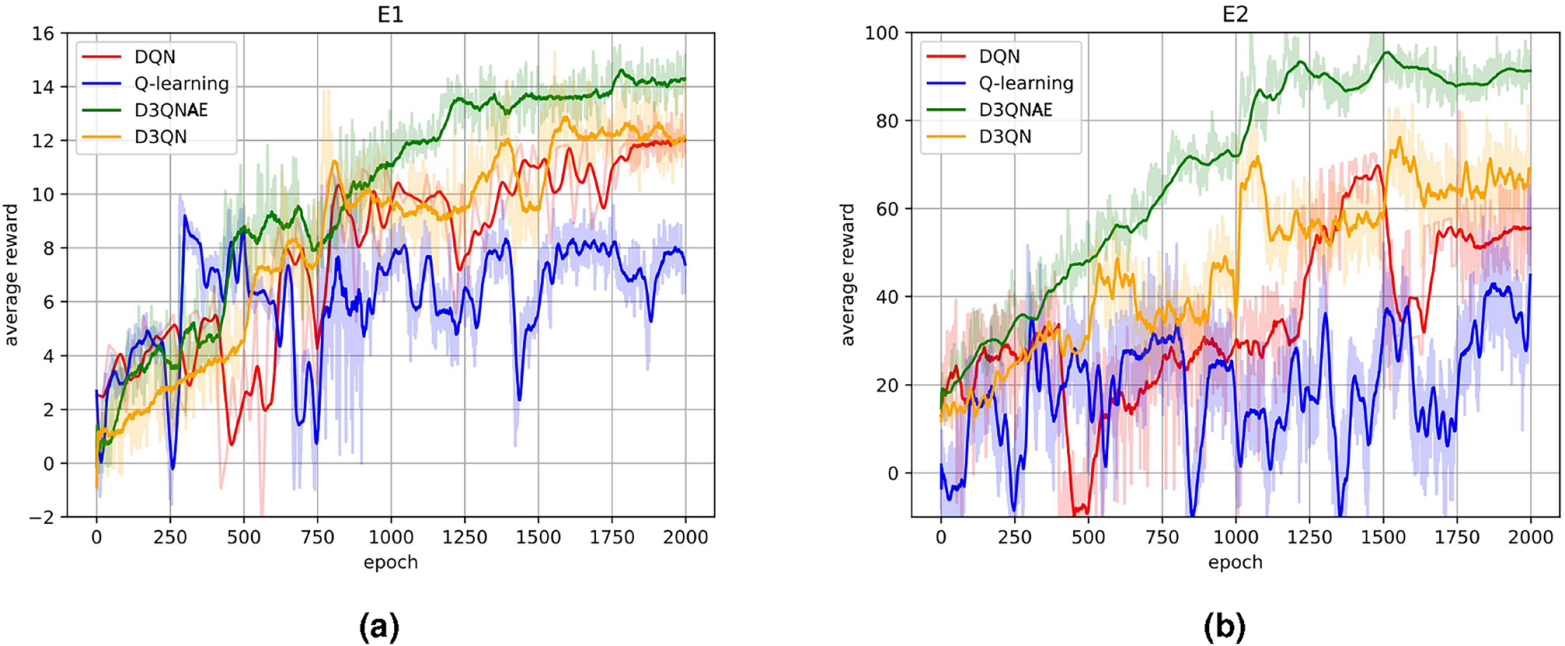
Comparison of reward and punishment function reward values during scheduling processes among different algorithms.

Figure 4a shows the reconfiguration strategies generated by D3Q-NAE, DQN, and other algorithms for solving continuous fault problems in Environment E1, where 15 task applications are deployed in the integrated electronic system. Compared to the algorithm performance in the single fault injection experiment, the solution space for continuous fault reconfiguration problems is significantly larger, and all algorithms exhibit more pronounced oscillations when generating reconfiguration strategies. The traditional tabular method, Q-learning, shows poor global optimal solution search capabilities, with values below 0 indicating infeasible solutions. The Q-learning algorithm fails to converge within the experimental iteration rounds, resulting in low-quality reconfiguration strategies. The D3QN and DQN algorithms, which incorporate neural networks, show a more pronounced upward trend but converge slowly, eventually outputting stable solution strategies. The D3QNAE algorithm exhibits a significant upward trend and has good local optima escape capabilities, converging faster.

Figure 4b presents the training results of generating reconfiguration strategies for the E2 environment with 100 task applications under continuous fault injection tests. Under continuous faults, both the D3QN and DQN algorithms can optimize their networks after training iterations to obtain good-quality feasible solutions. However, compared to solving smaller-scale problems, the oscillations are more pronounced, making it difficult to ensure the stability of the solution strategies. The Q-learning algorithm, in the initial iterations, can generate better-quality feasible solutions compared to the D3QN and DQN algorithms simultaneously, but it struggles to continue optimizing to generate high-quality solution strategies in subsequent iterations. In the large-scale system reconfiguration experiment iteration rounds, it fails to converge. Compared to the single fault injection experiment in the same environment, D3QNAE converges later but shows a relatively smooth curve compared to the D3QN and DQN algorithms. It can find high-quality feasible solutions within a shorter number of iterations and converge quickly after obtaining the feasible solution.

The comparison of algorithm solution performance primarily focuses on the time taken by each algorithm to converge and obtain a stable, feasible solution for the first time when the integrated electronic system deploys 15, 100, and 500 task applications, respectively. For each algorithm in the same environment, the time to obtain the first solution is recorded, and the average of 100 such times is taken as the final solution time for that algorithm.

**Table 3:** Solution time for learning algorithms in different environments.

Algorithms	E1_15	E2_100	E3_500
Q-learning	2h18m	>12h	>12h
DQN	55m20s	10h41m	>12h
D3QN	23m23s	7h20m	>12h
D3QNAE	15.58s	14m33s	35m20s

As shown in Table 3, under continuous fault conditions, only the D3QNAE, D3QN, and DQN algorithms that incorporate neural networks can solve for a stable, feasible solution within 1 hour. The traditional tabular method, Q-learning, requires more than two hours to solve, indicating that introducing neural networks enhances the algorithm’s generalization ability for higher-dimensional complex problems, improving the solution efficiency in complex scenarios. D3QNAE leverages its advantages by utilizing an agent’s adaptive exploration strategy based on the average sequence of cumulative rewards, which better balances exploration and exploitation, making the algorithm more robust, stable, and efficient in learning.

**Fig. 5:**
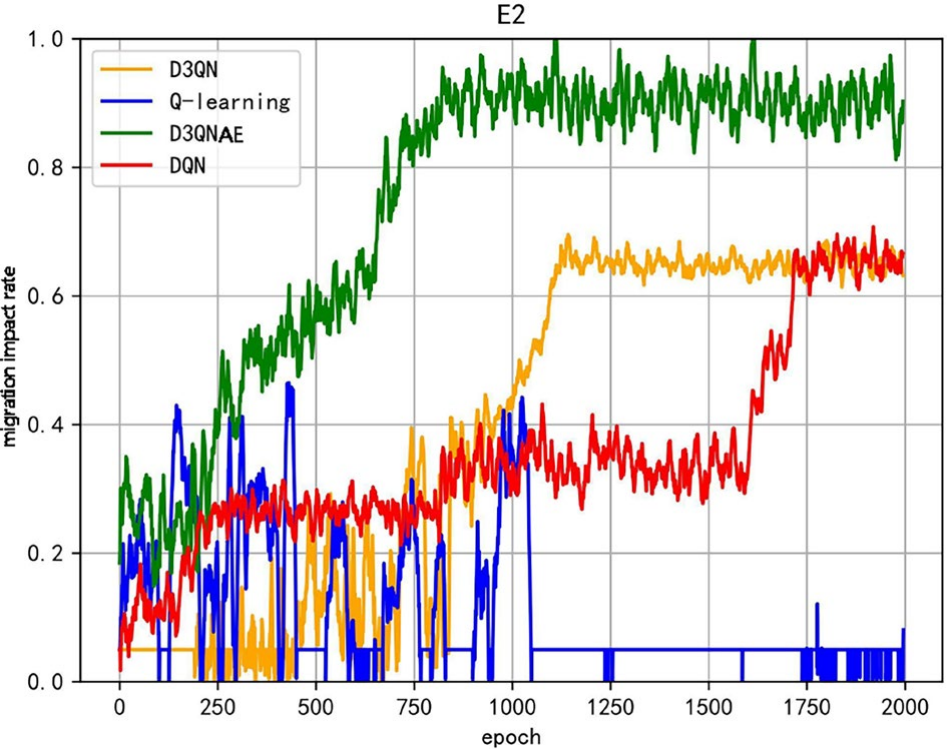
Comparison chart of migration impact rates.

In addition to the existing load balancing and reconfiguration recovery time metrics, the model evaluation indicators considered in our proposed algorithm include a migration impact rate aimed at improving the retention rate of container instances hosting critical task software during reconfiguration. Figure 5 shows experimental comparisons of the iterative performance of the migration impact rate of reconfiguration strategies generated by different algorithms, taking the E2 environment as an example.

As seen in Fig. 5, during continuous faults, when system resources decrease rapidly and there are a large number of faulty software, in the early to mid iterations, the migration impact rates for the D3QNAE, D3QN, DQN, and Q-learning algorithms are 1, 0.65, 0.65, and 0, respectively. A value of 0 indicates that only D3QNAE can generate a viable reconfiguration strategy within a limited time. D3QN and DQN algorithms complete the migration of all software but can only do so sequentially based on internal priorities, resulting in significant sacrifices. The Q-learning algorithm fails to generate an effective, feasible solution that meets system constraints and is unable to complete the reconfiguration. The data in the table shows that under the same number of training iterations, the D3QNAE algorithm demonstrates a stronger ability to generate reconfiguration strategies compared to other algorithms and has more advantages in simultaneously optimizing multiple objectives, especially the migration impact rate.

In summary, based on the comparative experiments of algorithm learning performance and the results of algorithm solution performance testing, it can be concluded that when the system is tasked with generating reconfiguration strategies for continuous faults, the proposed D3QNAE algorithm is still able to find a well-performing feasible solution in a relatively short time, and gradually optimize multiple objective optimization indicators, particularly the migration impact rate. Compared to other algorithms, D3QNAE exhibits significant advantages regarding solution effectiveness.

## Discussion

To further validate the solution performance of the proposed D3QNAE algorithm and set it apart from existing traditional solution methods, a comparative analysis is conducted against mathematical solution methods based on Finite State Machines (FSM), heuristic algorithms based on Genetic Algorithm (GA), and Multi-Objective Particle Swarm Optimization (MOPSO). The experiments compare the generation time of reconfiguration strategies for continuous fault scenarios under the same task application deployment scenario.

**Fig. 6:**
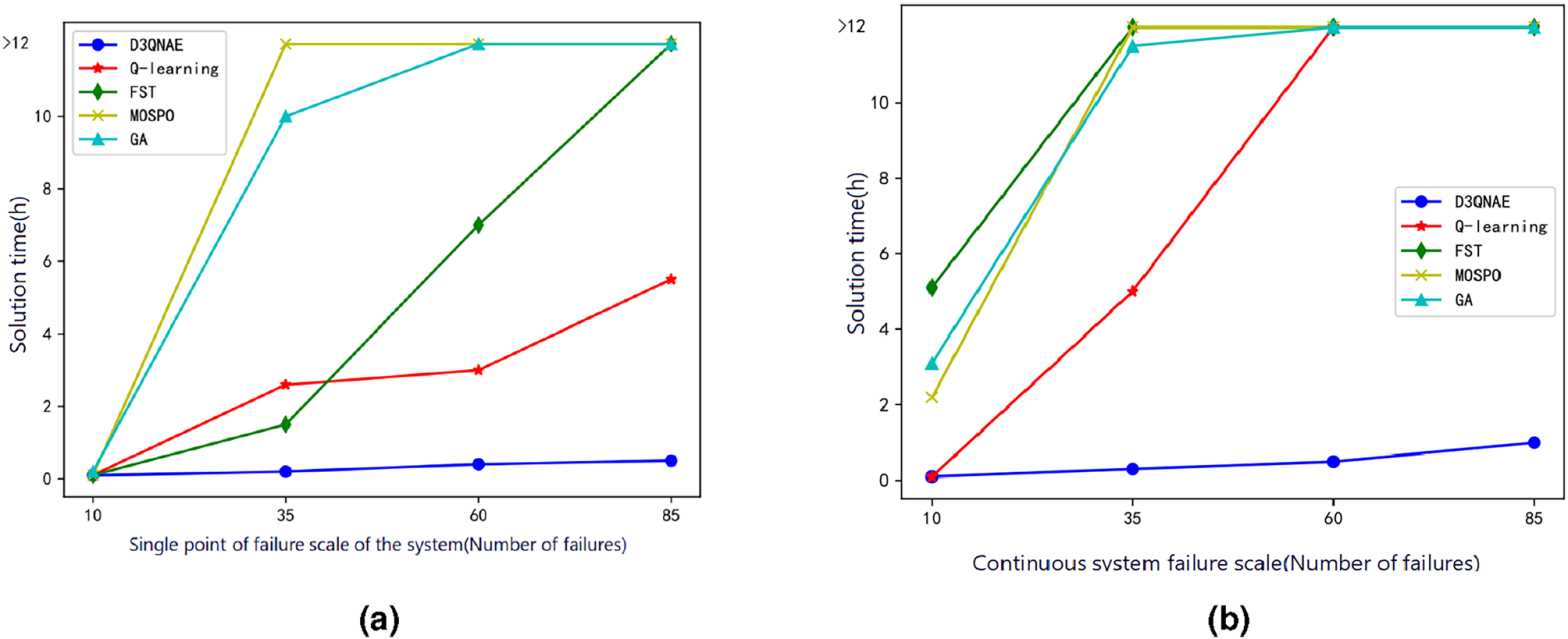
Comparison of solution times for fault experiments in different environments.

As shown in Fig. 6, the heuristic algorithms GA and MOPSO take relatively long solution times. The mathematical optimization method based on FST has shorter solution times for generating reconfiguration strategies for small-scale single-fault scenarios than other algorithms. These solution times decrease as the environmental complexity increases. However, when the scale of the single-fault reconfiguration problem exceeds 85, it becomes difficult to find a feasible solution within a limited time. In cases of larger-scale continuous faults, the algorithm may even crash. In contrast, while Q-learning based on reinforcement learning can handle continuous fault situations, its solution time increases with the complexity of the environment. The proposed D3QNAE algorithm, on the other hand, introduces a dynamic adaptive exploration mechanism based on the average sequence accumulation, enabling the agent to more effectively balance exploration and exploitation, resulting in improved algorithm performance. Within the same training time, it achieves better performance, and the solution time for the problem of triggering network timing scheduling only increases linearly with the increase in the size of the solution message without a significant relationship to the complexity of the environment.

**Table 4:** Comparison of optimal solutions in failure experiments for algorithms.

Experiments	Reconfiguration status	Algorithm						
		D3QNAE	D3QN	DQN	Q-learning	FSM	MOSPO	GA
Single failure	Reward value	0.952482	0.948426	0.948716	0.927730	0.780584	0.824156	0.826660
	*LB*	0.868650	0.856329	0.863579	0.853429	0.801784	0.828163	0.850640
	*RT*	0.940000	0.940000	0.940000	0.940000	0.820000	0.840000	0.840000
	*MI*	1.000000	1.000000	1.000000	1.000000	1.000000	1.000000	1.000000
Continuous failure	Reward value	0.852148	0.847617	0.836083	0.623659	0.497757	0.573775	0.511831
	*LB*	0.821581	0.812830	0.821581	0.772401	0.734613	0.788805	0.734161
	*RT*	0.920000	0.920000	0.860000	0.840000	0.600000	0.620000	0.600000
	*MI*	1.000000	1.000000	1.000000	0.818182	0.500000	0.666667	0.666667

As shown in Table 4, overall, the reconfiguration strategy generation algorithms that incorporate reinforcement learning produce strategies with better quality than traditional solution methods such as FSM and a series of heuristic methods like MOSPO and GA. Comparing the solution times, it is found that the FSM method is faster for small-scale problems, with a migration impact rate index of 1.0 for the generated reconfiguration strategies, indicating good performance in maintaining system functions. However, as the complexity of the problem increases, the algorithm is significantly affected by the problem scale, making it difficult to generate high-quality solutions. The series of heuristic algorithms GA and MOSPO perform weakly in terms of solution time, and as the solution space expands, they struggle to respond quickly to system fault reconfiguration demands. However, compared to the FSM algorithm, they exhibit advantages in solution quality. The integrated electronic system reconfiguration algorithm incorporating reinforcement learning demonstrates strong performance in solution quality, basically meeting the system migration impact rate requirements and effectively restoring system functions under fault conditions. Among them, the D3QNAE algorithm we propose, relying on the adaptive exploration strategy based on the agent’s average sequence accumulation, can find feasible solutions in a shorter time, even as the complexity of the system environment further increases. After rapid iterative exploration, it outputs relatively excellent reconfiguration strategies.W

In summary, based on the above experiments, finding a feasible solution requires a relatively large exploration range under the complex functional deployment of integrated electronic systems. As shown in Tables 3 and  4, experimental results demonstrate that D3QNAE reduces the first feasible solution time by 34$\%$ compared to the best baseline (D3QN) in large-scale deployments (500-task scenarios), while achieving a 15.6$\%$ higher maximum reward value and a 100$\%$ migration impact rate under continuous faults. It improves convergence on top of the original exploration range, accelerates the solution speed, and provides a solution for solving such problems.

## Conclusions

We propose an embedded container reconfiguration method based on the D3QNAE for the new generation of aviation-integrated electronic systems utilizing container technology. By incorporating an adaptive exploration strategy based on average sequence accumulated reward, our approach significantly enhances the ability to generate efficient and stable reconfiguration strategies in complex environments. Experimental results show that the D3QNAE algorithm can rapidly find superior solutions under both single and continuous fault conditions, demonstrating better global search capability and solving efficiency in large-scale task deployments compared to existing algorithms. Moreover, the introduced migration impact rate metric improves the retention of container instances hosting critical task software, ensuring system functionality and stability. Overall, this study provides an effective solution for addressing reconfiguration challenges in next-generation aviation embedded systems.

## Data Availability

The datasets generated and analysed during the current study are not publicly available due to the confidentiality of the experimental data, but are available from the corresponding author on reasonable request.
